# Reading is for girls!? The negative impact of preschool teachers' traditional gender role attitudes on boys' reading related motivation and skills

**DOI:** 10.3389/fpsyg.2015.01267

**Published:** 2015-08-24

**Authors:** Ilka Wolter, Edith Braun, Bettina Hannover

**Affiliations:** ^1^Department of Education and Psychology, Freie Universität BerlinBerlin, Germany; ^2^International Centre for Higher Education Research at Universität KasselKassel, Germany

**Keywords:** preschool teachers, gender role attitude, boys' underachievement, precursors of reading skills, reading related motivation, reading skills, gender stereotypes

## Abstract

According to gender stereotypes, reading is for girls. In this study, we investigated the role of preschool teachers in transmitting such gendered expectations. We suggest that boys are less motivated to read in preschool, and less competent in reading 1 year later in primary school, if their preschool teacher holds a traditional gender role attitude than if the teacher has egalitarian beliefs. In 135 independent dyads of a female preschool teacher (*N* = 135) and one boy (*n* = 65) or one girl (*n* = 70) we measured teacher's gender role attitude, child's reading related motivation as well as precursors of reading skills in preschool, and child's reading skills at the end of first grade in primary school. As expected, the more traditional preschool teachers' gender role attitude was, the weaker was boys' motivation to (learn to) read while girls' motivation was unrelated to teachers' gender role attitude. In either gender, motivation in preschool predicted reading skills at the end of first grade.

## Introduction

Reading skills are essential for individuals to gain an understanding across subject domains in school and hence are an important predictor of their future socioeconomic success (e.g., Duncan et al., 2007; Ritchie and Timothy, 2013). Sadly, boys have consistently been found to be less competent readers than girls, across different countries and languages (e.g., in PIRLS 2011, reported in Mullis et al., 2012).

The goals of this research are as follows: We wanted to test the assumption that preschool teachers have an influence on gender typing in children's development of reading related skills. While the causes for boys' underachievement in reading are manifold (see Lynn and Mikk, 2009; Martin and Ruble, 2010, for reviews), in this research we want to focus on one potentially relevant factor: the gender role attitude of the preschool teacher. We predicted that preschool teachers who share traditional views with respect to gender roles shape children's attitudes and behaviors in a gender typed manner: Boys whose preschool teacher has a traditional gender role attitude should be less motivated to read than boys with a preschool teacher holding more egalitarian beliefs, with this lowered motivation in turn having a negative impact on boys' reading achievement in primary school 1 year later.

While reading related skills have extensively been studied in samples of children in (mostly later) primary school grades and secondary school (see Morgan and Fuchs, 2007, for a review), research on emergent readers attending preschool is scarce. Precursors of reading competence, such as phonological awareness or phonological recoding in lexical access, start to develop long before school entry, i.e., during the preschool years (see Townsend and Konold, 2010; Hulme and Snowling, 2013, for reviews), with some studies reporting gender differences to already appear in these early precursor competences (e.g., Lundberg et al., 2012; Wolter et al., 2014), continuing into girls outperforming boys in their reading achievements in first and second grade of primary school (e.g., McCoach et al., 2006; Niklas and Schneider, 2012).

Becoming a skilful reader not only requires precursor competences but also a sufficiently strong motivation to (learn to) read (see Gambrell and Gillis, 2007, for a review). Boys typically describe their motivation to (learn to) read as less strong than girls do (e.g., for first, second, and third graders: Guay et al., 2010; for fourth, fifth, and sixth graders: McGeown et al., 2012). This is particularly worrisome as reading related motivation has been found to covary with reading skills in primary school students (see Morgan and Fuchs, 2007, for a review): The motivation to engage in reading related activities predicts the amount of reading (e.g., Cox and Guthrie, 2001) which in turn predicts growth in reading competence during the primary school years (e.g., Guthrie et al., 2007; Taboada et al., 2009). To summarize, boys' lower reading attainments are related to a weaker motivation to read.

Against this background, in addition to investigating the potential influence of preschool teachers' gender role attitudes, we wanted to test (a) whether the relation between reading related motivation and reading skills can already be found in preschool aged children, (b) whether reading related motivation captured as early as in preschool predicts the development of reading skills during children's first year in primary school, and (c) whether girls and boys already differ in their reading related motivation and precursors of reading skills before school entry.

### Reading related gender stereotypes

Boys' lower attainments and motivation in reading seem to reflect gender stereotypes according to which reading is for girls. Developmental research shows that children start to acquire gender stereotypes as early as 2–3 years old, with that knowledge accumulating until about school entry (e.g., Trautner et al., 2005; Banse et al., 2010). Dwyer (1974) found that already in second grade, children endorse reading related gender stereotypes: throughout the school years until grade 12, girls and boys were inclined to describe reading as a feminine activity. Pottorff et al. (1996) found children from grades 2, 4, 6, and 8 to associate reading books with mothers rather than fathers and to consider girls as better able to read books than boys. Similarly, Millard (1997) interviewed seventh graders and found them to associate their mothers rather than their fathers with reading activities at home and with being taught to read. Martinot et al. (2012) found boys and girls from fifth grade to believe that people conceive of girls as superior to boys in reading. Several studies found secondary school students to conceive of subjects related to reading as “for girls” (e.g., Hannover and Kessels, 2002; Colley and Comber, 2003; Plante et al., 2009; Steffens and Jelenec, 2011).

### Transmission of gender stereotypes by preschool teachers

An extant body of research attests to stereotypes operating in a self-fulfilling manner (e.g., Hamilton et al., 1990): Expectations accompanying a stereotype, e.g., that reading is for girls, trigger a chain of events that finally result in its confirmation, in our case that boys are less motivated to learn to read and therefore, finally, become less skillful readers. The many ways in which children acquire gender stereotypes have not yet been fully identified. One important mechanism of transmission are significant others, such as parents, preschool or school teachers (see Schoon and Eccles, 2014, for a review). They exert their influence via social modeling, via the expression of their own beliefs about the genders, or via direct socialization practices.

First, significant others serve as role models via their own gendered behaviors. For instance, a female teacher engaging in both female (e.g., cooking and baking) and male stereotyped activities (e.g., playing soccer) can be assumed to foster less gender typed attitudes and behaviors in the children of her preschool group than a female teacher offering female stereotyped activities only (cf. Wolter et al., 2014). Indirect evidence for this assumption comes from research showing that children from mothers being employed in the workforce hold less traditional gender role attitudes and that girls with employed mothers are more inclined to strive for an occupation than girls whose mothers stay at home (e.g., Willetts-Bloom and Nock, 1994; Jackson and Tein, 1998).

Second, significant others influence children's assumptions about gender roles by expressing their own gendered expectations. Imagine, for instance, a teacher saying in front of her preschool group of children that the girls probably want to stay inside while the boys would probably prefer to play outside. Indirect evidence for such a transmission of gender stereotypes comes from research on parents' or teachers' gendered ability related expectations (e.g., Tiedemann, 2000; Rouland et al., 2013; see Jacobs et al., 2005, for a review). For instance, Upadyaya and Eccles (2014) found primary school teachers to rate boys as higher in math ability than girls, and girls as putting more effort into reading than boys, with these perceptions in turn predicting children's ability self-concepts in math and reading. Similarly, Retelsdorf et al. (2015) found teachers' reading related gender stereotypes (do boys or girls read better, read more, and have more fun reading?) to favor girls and, when measured at the beginning of grade 5, to predict boys' more negative reading self-concept at the end of grade 6 (controlling for previous self-concept and reading attainments). Other research has shown that gender related attitudes and stereotypes are transmitted across generations within families (e.g., Carlson, 2011; Endendijk, 2013; Farré and Vella, 2013; Hess et al., 2014). For instance, investigating 244 German families, Hess et al. (2014) found that the more traditional fathers' gender role attitudes were, the more traditional were the attitudes of their sons and daughters 5 years later.

Third, significant others also directly shape gendered behaviors by either reinforcing, ignoring or punishing children's affiliation toward or engagement in different activities, or children's expression of certain self-beliefs (e.g., their ability self-concepts). For instance, a preschool teacher may be more inclined to comfort a girl crying than a boy crying by giving the child a cuddle. Evidence for such means of transmission comes from Lytton and Romney's (1991) classic meta-analysis: they found systematic differences in parents' socialization practices toward their sons vs. daughters in that they tended to encourage gender-typed activities, i.e., different activities depending on their child's gender. To give one example: Simpkins et al. (2005) found mothers of sons to report more encouragement and provision of materials for computer, math, and science related activities than mothers of daughters.

### Preschool teachers' gender role attitudes as indicator of gendered socialization practices

As explained above, we assume that preschool teachers influence children's gender development via social modeling, expression of expectations about the genders, and via direct socialization practices. To capture the extent to which teachers are inclined to—directly or indirectly—support gender typed attitudes and behaviors in their preschool group of children via these mechanisms we measured their gender role attitudes.

Individuals differ in their gender role attitudes (also: gender role orientation, gender role beliefs, gender role ideology), i.e., in the extent to which they have internalized societal norms about the traditional division of labor between the genders—with men being the breadwinners and women taking care of household and children—and societal expectations according to which behaviors differ in their appropriateness or desirability depending on the actor's biological sex. While individuals with a *traditional gender role attitude* differentiate traits, attitudes, and behaviors as either more typical/desirable for males or for females and, accordingly, endorse the gendered distribution of labor, individuals with an *egalitarian gender role attitude* reject differences between the genders as described by gender stereotypes and disapprove of individuals being assigned to different tasks and resources based on their biological gender. Many different instruments measuring gender role attitudes have been developed (see Frieze and McHugh, 1998, for a review on scales in English; for scales in German: Krampen, 1983; Athenstaedt, 2000).

We expected that teachers' gender role attitudes would systematically relate to the socialization practices they deploy in their preschool group of children. Indirect support for this assumption comes from a study by Cahill and Adams (1997) who found preschool teachers' attitudes toward adult gender roles (e.g., work roles, parental responsibilities) to strongly correlate with their attitudes toward children's gender roles, and child rearing practices (e.g., encouragement of gendered behaviors). Teachers who expressed non-traditional adult gender roles also endorsed non-traditional gender role childrearing practices.

Preschool teachers' gender role attitudes should translate into their treating children in a more or less gender stereotyping manner, such that teachers with a traditional gender role attitude foster gender differences, here in reading motivation, more than teachers with egalitarian beliefs. Indirect evidence for this assumption comes from a study by Kingsbury (2012). Mothers of preschool-aged girls and boys were asked to report their gender role attitudes and to describe how they would react if their child displayed extremely aggressive or shy behaviors. As expected, particularly mothers with traditional gender role attitudes were inclined to respond with negative emotions in response to children's gender incongruent behaviors (i.e., shyness in boys, aggressiveness in girls). Further support for our assumption that preschool teachers with traditional gender role attitudes treat children in a more gender stereotyping manner than teachers with egalitarian beliefs comes from the study by Hess et al. (2014) which, however, investigated a sample of older children. The authors did not only find fathers' gender role attitudes (measured when the child was about 14 years old) to directly impact the child's gender role beliefs 5 years later, but also indirectly via parenting practices (as reported by the child) which were the more gender typed the more traditional fathers' gender role attitudes had been.

### Hypotheses and study overview

The goals of our study were two-fold:

- First, we wanted to identify a potential impact of preschool teachers' gender role attitudes on children's skill development in reading. To that end we measured teachers' gender role attitudes and linked it with data on children's reading related motivation.
  Our hypothesis was as follows: The preschool teacher's gender role attitude has a differential effect depending on the child's gender in that: (1) the more traditional the teacher's gender role attitude is, the less motivated boys are to learn to read while (2) for girls, the more traditional the teacher's gender role attitude is, the more motivated girls are to learn to read, or their motivation is unrelated to their teacher's gender role attitude.
- Second, we wanted to investigate the interrelatedness between motivation to read and reading skills in a sample of preschoolers. Available reading motivation instruments typically target older groups of children and assume that children can read (see Sperling et al., 2013, for a review). The few studies investigating preschoolers have typically used parents' (e.g., Yeo et al., 2014) or teachers' (e.g., Lepola, 2004; see Morgan and Fuchs, 2007, for a review) judgements on the child's reading motivation. As teacher judgements on children's reading motivation may be biased by their gender role attitudes, in our study we wanted to use children's self-reports. We captured the child's reading related motivation in individual standardized interviews, based on a set of seven questions tailored for our age group of preschoolers. In addition, we measured precursors of reading skills using standardized tests. Reading related motivation and precursors of reading skills were measured during the child's last year in preschool. Children were followed up after transition to primary school and tested for their reading skills at the end of first grade.

Our hypotheses were as follows: Reading related motivation in preschool is correlated with precursors of reading skills in preschool. Reading related motivation in preschool predicts reading skills at the end of first grade of primary school (direct effect). Precursors of reading skills impact later reading skills via reading related motivation in preschool (indirect effect).

## Methods

### Sample

Our sample consisted of 135 dyads of a female preschool teacher (*N* = 135) and one boy (*n* = 65) or one girl (*n* = 70) from their group of children. To obtain independent child-teacher dyads, only one dyad was drawn from randomly selected preschool groups in Berlin (for a more detailed description on the sampling strategy see Wolter et al., 2014). All parents were asked for their written consent prior to the first data collection. We only included children whose parents and teachers had given their consent to take part in the study. Teachers and parents were informed that participation was voluntary, and that they could opt-out at any time^1^.

Every child and every teacher was investigated in an individual session. Children were first tested during their last 2 months of preschool (t1), and followed up at the end of first grade (t2), on average 13 months after the first measurement, Min = 10 months, Max = 14 months. At t1, children were aged *M* = 71.4 months, *SD* = 3.3, range = 65–78 months. From t1 to t2, our sample was reduced by 28 children (in most cases because they could no longer be reached after transition to primary school). Therefore, our sample size at t2 was *N* = 106 children. The preschool teachers were investigated only once, at t1. They were aged *M* = 43.51 years, *SD* = 8.15 years, range = 23–58 years. At t1, children had been with this particular teacher in their preschool group between 2 and 65 months, *M* = 2.4 years, *SD* = 19 months. On average the children spent about 8 h per day in preschool, *M* = 7.27, *SD* = 0.94, range 5–10 h.

The mean level of socioeconomic background of the children's families, as measured at t1 and operationalized by the HISEI (Highest International Socio-Economic Index of Occupational Status; Ganzeboom et al., 1992), was *M* = 57.73, *SD* = 14.75, and thus somewhat higher than in representative samples of German families (e.g., the German sample of PISA 2009, Klieme et al., 2010, p. 235: *M* = 48.9, *SD* = 15.6).

### Data analysis

Although the sample basically consisted of independent child-teacher dyads, from 16 preschools more than one dyad was drawn—always from a different group led by a different teacher. As we did not analyse variables from different levels simultaneously, however, there was no need for multilevel-modeling (e.g., Hox, 2002). As we measured different variables in teachers (gender role attitudes) and children (motivation and skills), the data are also independent within dyads, i.e., there is no need to correct for non-independence (e.g., Kenny, 1996). According to MacKinnon (2008), when testing the significance of indirect paths, common methods of significance testing (e.g., joint significance test; Baron and Kenny, 1986) are somewhat inaccurate due to the non-normality of products of two paths. We therefore evaluated the confidence intervals of indirect paths and applied a bootstrapping method (using 1000 bootstrap samples) to correct for potential sample-bias.

To test our hypotheses we conducted a path model using the statistical software *MPlus* 5.1 (Muthén and Muthén, 1998–2010). All predictor variables were entered as grandmean-centered variables into the regression analyses. Missing values were treated as missing at random by using full information maximum likelihood estimators.

### Research instruments

#### Preschool teachers' gender role attitudes

To measure gender role attitudes, preschool teachers were asked to fill in the scale by Athenstaedt (2000) at t1. The unidimensional scale consists of 29 items (five-point answering scales: 1 = strongly disagree, 5 = strongly agree) describing either traditional (e.g., “Both boys and girls should undertake household chores”; “For a good first impression a neat appearance is more important for a woman than for a man”) or egalitarian views toward gender roles (e.g., “Women are as qualified as men for a leadership position in an engineering company”; “When it comes to politics, men should listen to women to a greater extent”; “A higher number of male preschool teachers would be pleasing”).

Out of the 29 items, the 12 items describing an egalitarian view were recoded. Afterwards, we calculated a mean score for every teacher, with higher scores indicating more traditional gender role attitudes. The scale reached Cronbach's α = 0.703. On average, teachers responded below the scale's mean, *M* = 1.94, *SD* = 0.34, range = 1.14–2.86, indicating rather egalitarian gender role attitudes.

#### Reading related motivation in preschool

At the end of preschool (t1) we measured reading related motivation via seven items regarding children's liking for different reading related tasks (“How much do you like to learn new rhymes or poems or songs?”; see Appendix; cf., Bachmann and Burock, 2008). Children had to respond on scales consisting of three “smiley” faces: one with a big smile, (3 = I like it very much), one with a slight smile 2 = I like it, and one with a neutral expression (1 = I don't like it much). The scale reached an internal consistency of Cronbach's α = 0.70, with an overall mean of *M* = 2.16, *SD* = 0.48.

#### Precursors of reading skills in preschool

Precursors of reading skills were measured at the end of preschool (t1) using the “Bielefelder Test zur Früherkennung von Lese-Rechtschreibschwäche” (Jansen et al., 2002; *BISC; Bielefelder Screening for Early Detection of Dyslexia*). The BISC taps into four different phonological information processing skills (phonological awareness, phonological recoding in lexical access, phonetic recoding in working memory, visual spatial attention regulation); using eight different tasks (e.g., repeating pseudo-words, finding rhyming words, naming the colors of uncoloured or incongruent objects, segmenting syllables, matching phonemes and words). Children achieved a mean sore of *M* = 67.90, *SD* = 7.47, out of a possible 82 points. Internal consistencies for the subscales were ranging between Cronbach's α = 0.63 and 0.85.

#### Reading skills in primary school

To investigate children's reading skills at the end of first grade in primary school (t2), we used the “Wuerzburger Leise Leseprobe,” a one scale speed-test of silent reading applicable for first to fourth graders (Küspert and Schneider, 1998). The test consists of 140 tasks children have to work on. In our sample, reliability of the scale was very good (Kuder-Richardson formula for dichotomous items: 0.96). The children mastered between 13 and 106 tasks, *M* = 42.34, *SD* = 18.09. Test scores of our sample are comparable to norm values for this age group (norm sample from item development: *N* = 646, *M* = 42.82, *SD* = 17.15; *t*_(779)_ = 0.0817, n.s., *d* = 0.01).

## Results

### Gender differences in reading related motivation and (precursors of) reading skills

In a first step, we tested whether our measures of reading related motivation and precursors of reading skills were invariant across the genders by comparing the measurement models via a confirmatory factor analysis approach. For reading related motivation, the model fit, χ^2^ = 44.979, *df* = 39, *p* = 0.236; CFI = 0.927, TLI = 0.921, RMSEA = 0.048, 90%CI 0.000–0.102; SRMR = 0.107, and an insignificant χ^2^-difference to the prior metric model, Δχ^2^ = 7.709, *df* = 4; *p* = 0.102, confirmed partial scalar invariance, such that the prerequisite condition for latent mean comparisons was met. The only restriction from total scalar invariance was the free estimate for two items' intercepts (Items 1 and 7). Similar findings were obtained for precursors of reading skills: the model fit, χ^2^ = 36.023, *df* = 39, *p* = 0.606; CFI = 1.00, TLI = 1.05, RMSEA = 0.000, 90%CI 0.000–0.076; SRMR = 0.132, and an insignificant χ^2^—difference to the prior metric model, Δχ^2^ = 9.103, *df* = 6, *p* = 0.168, confirmed partial scalar invariance. The only restriction from total scalar invariance was the free estimate of two subscales' factor loadings (i.e., “Laut-zu-Wort” and “Silben-Segmentieren”).

These findings suggest that the measurement models for boys and girls are comparable, such that differences in latent mean values can be interpreted as actual gender differences. Girls, *M* = 2.25, *SD* = 0.42, indicated higher reading related motivation than boys, *M* = 2.06, *SD* = 0.52, at the end of preschool, *b*_(*diff*)_ = 0.536, *SE* = 0.266; *z* = 2.015, *p* = 0.044. Also as expected, girls obtained significantly higher scores, *M* = 69.67, *SD* = 5.16, than boys, *M* = 65.99, *SD* = 8.94, in precursors of reading skills at the end of preschool, *b*_(*diff*)_ = 0.597, *SE* = 0.296, *z* = 2.018, *p* = 0.044.

Further, we tested gender differences in reading skills in primary school. As reading skills were measured by a speed test with a single value (sum score), as indicator of participants' actual skills, measurement invariance is assured. As expected, boys, *M* = 38.32, *SD* = 15.63, were outperformed by girls, *M* = 46.16, *SD* = 18.74, in their reading skills at the end of first grade in primary school, *t*_(133)_ = 2.63, *p* < 0.01, *d* = 0.46.

### The impact of preschool teachers' gender role attitudes on children's reading related motivation

To test our hypothesis referring to the impact of motivation on later reading achievement, we regressed children's reading skills at the end of first grade on reading related motivation in preschool. To further test our hypothesis that preschool teachers' gender role attitudes would impact the development of children's reading related motivation and skills, we regressed children's reading related motivation in preschool on their precursors of reading skills and their preschool teacher's gender role attitude. As we expected preschool teachers' attitudes to be differentially related to reading motivation in boys vs. girls, we also included the term for the statistical interaction of teachers' gender role attitudes and children's gender into the analysis. In addition, as there was considerable variation in the time span that the children in our sample spent with their preschool teacher, we included it as a control variable. As previous research found children's reading attainments to depend on their families' socioeconomic background (e.g., Mullis et al., 2012), we included HISEI as a control variable for children's precursors of reading skills in preschool. Furthermore, we also included the time children spent in preschool per day as an additional control variable for precursors of reading skills.

Zero-order correlations (i.e., Pearson's correlation coefficients) among the predictor variables were as follows: As expected, children's reading related motivation was correlated with precursors of reading skills in preschool, *r* = 0.28, *p* < 0.01. Socioeconomic family background neither correlated with reading related motivation, *r* = −0.08, n.s., nor with precursors of reading skills, *r* = 0.13, n.s.

The model fit of the path model was χ^2^ = 18.037, *df* = 12, *p* = 0.115; CFI = 0.902, TLI = 0.828, RMSEA = 0.061, 90%CI 0.000–0.115; SRMR = 0.057. Results (see Table 1) revealed, as expected, that reading related motivation in preschool predicted reading skills 1 year later, *b* = 8.38, *SE* = 3.49, β = 0.21, *p* < 0.05 *d* = 0.80. Moreover, the expected two-way interaction was observed, indicating that teachers' gender role attitudes had a differential effect on reading related motivation in boys vs. girls, *b* = 0.62, *SE* = 0.21, β = 0.34, *p* < 0.01, *d* = 0.98.

**Table 1:** **Path model for (a) reading skills as predicted by reading related motivation, and (b) reading related motivation as predicted by children's precursor of reading skills, preschool teacher's gender role attitudes, and interaction of child's gender and preschool teacher's gender role attitudes, with time spent with preschool teacher controlled for, and (c) children's precursor of reading skills with socioeconomic background (HISEI) and time spent in preschool per day controlled for**.

	***b***		***SE***	**β**	**d**
**READING SKILLS (t2) ON**					
Intercept	43.49	^***^	1.67		
Reading related motivation	**8.38**	^*^	3.49	0.21	0.80
	*R*^2^	0.045			
**READING RELATED MOTIVATION (t1) ON**					
Child's gender	**0.14**	^*^	0.07	0.16	0.67
Time spent with teacher (months)	0.00		0.00	0.05	0.20
Precursors of reading skills	**0.02**	^***^	0.01	0.29	1.17
Teacher's gender role attitude	−0.11		0.11	−0.09	−0.34
Interaction child's gender ^*^ teacher's gender role attitude	**0.62**	^**^	0.21	0.24	0.98
	*R*^2^	0.198			
**PRECURSORS OF READING SKILLS (t1) ON**					
Child's gender	**2.33**	^*^	1.03	0.19	0.75
Socioeconomic background (HISEI)	**0.07**	^+^	0.04	0.17	0.60
Time spent in preschool per day (hours)	0.66		0.64	0.13	0.34
	*R*^2^	0.072			

*** *p < 0.001*,

** *p < 0.01*,

* *p < 0.05*,

+ *p < 0.10; predictor variables grandmean-centered; significant coefficients are in boldface*.

Further, our findings showed that the time the child had already been with the respective teacher was irrelevant for the prediction of reading related motivation, *b* < 0.01, *SE* < 0.01, β = 0.05, n.s. Results also showed that children's socioeconomic background was related to precursors of reading skills in preschool, however, only marginally significantly so, *b* = 0.07, *SE* = 0.04, β = 0.17, *p* = 0.057, *d* = 0.63. Precursors of reading skills in preschool and reading related motivation were significantly correlated, *b* = 0.02, *SE* = 0.01, β = 0.29, *p* < 0.001, *d* = 1.17.

*Post-hoc Probing (cf. Aiken et al., 1991)*. The results from *post-hoc* probing (i.e., simple-slopes analyses) showed that consistent with our hypothesis, the more traditional preschool teacher's gender role attitudes were, the less boys—but not girls—were motivated to learn to read in preschool (boys: *b* = −0.42, *SE* = 0.16, β = −0.33, *p* < 0.01, *d* = −0.89; girls: *b* = 0.20, *SE* = 0.14, β = 0.16, n.s.).

**Figure 1:**
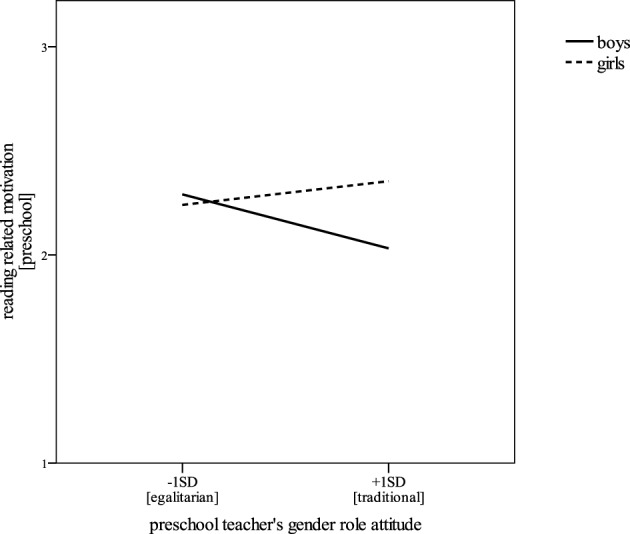
**Reading related motivation of girls and boys in preschool as predicted by their preschool teacher's gender role attitude**.

To further investigate this differential effect, we conducted additional simple-difference tests to compare the reading related motivation of children whose preschool teachers had either quite traditional (1 *SD* above the scale's mean) or quite egalitarian attitudes toward gender roles (1 *SD* below the scale's mean) (see Figure 1). As expected, simple difference-tests revealed gender differences in reading related motivation for those children who were with a rather traditional preschool teacher, *b* = 0.35, *SE* = 0.10, β = 0.40, *p* < 0.001, *d* = 1.11, but not for children whose teacher was rather egalitarian, *b* = −0.07, *SE* = 0.09, β = −0.08, n.s. In fact, in groups with an egalitarian teacher, boys were as motivated to read as girls in preschool.

*Indirect Effects*. To test the significance of the indirect paths we evaluated the confidence intervals of the expected indirect effect. The indirect effect of teacher's gender role attitudes on children's reading skills, mediated by children's reading related motivation, was significant for boys, *b* = −3.50, *SE* = 2.07, β = −0.07, *p* = 0.091, *d* = −0.56, but not for girls, *b* = 1.69, *SE* = 1.36, β = 0.03, n.s. Furthermore, though only marginally significant, children's precursor skills in preschool were linked to their later reading skills in primary school, with this effect again being mediated by children's reading related motivation, *b* = 0.17, *SE* = 0.09, β = 0.06, *p* = 0.070, *d* = 0.60.

To sum up, the results showed a differential impact of preschool teachers' gender role attitudes on boys' vs. girls' reading related skill development. The more strongly the preschool teacher endorsed traditional gender roles, the less were boys interested (to learn) to read in preschool and the lower were boys' reading skills at the end of primary school. In contrast, teacher's gender role attitudes neither had a substantial effect on girls' reading related motivation in preschool nor on their later reading skills in primary school.

## Discussion

In this research we wanted to find out whether preschool teachers play a role in children's gendered skill development. In a sample of 135 independent dyads of a female preschool teacher and one boy or one girl from their group of children we found that in fact, gender differences in reading related motivation were, to some extent, explained by teachers' gender role attitudes. Interestingly, our preschool teachers indicated rather egalitarian views on the gender roles, replicating what an earlier study found in teachers for preschool aged children in Turkey (Erden, 2004) and consistent with studies showing that teachers and teacher students hold more egalitarian views when they are female than when they are male (Lasonen and And, 1991; Togrol and Onur, 2000). The more traditional the gender role attitudes of the preschool teachers participating in our study had been, the less were boys motivated to (learn to) read while in preschool and the poorer they performed on a reading skill test one a year later in primary school. While boys and girls did not differ in the strength of their reading related motivation when with a preschool teacher with an egalitarian gender role attitude, boys were significantly less motivated to read when their teacher endorsed traditional gender beliefs. In contrast, girls' reading related motivation was the same, irrespective of how their preschool teachers thought about gender roles. This finding is in line with previous research: With reading being perceived as a female activity (e.g., Dwyer, 1974; Pottorff et al., 1996; Millard, 1997; Hannover and Kessels, 2002; Colley and Comber, 2003; Plante et al., 2009; Steffens and Jelenec, 2011; Martinot et al., 2012; McGeown et al., 2012), traditionally oriented preschool teachers should be less likely to encourage boys to engage in reading, as it is a “gender incongruent” activity. In contrast, both teachers with traditional and egalitarian gender role attitudes should consider reading an appropriate activity for girls, and thus not affect girls' reading motivation differently.

As research on the interrelatedness of reading related skills and motivation in preschoolers is scarce, we also wanted to investigate (a) whether the interrelatedness between reading motivation and reading related (precursor) skills which previous studies have reported for older groups of children can already be found before school start, (b) whether reading motivation in preschool forecasts reading skills in first grade of primary school, and (c) whether gender differences in reading related motivation and precursors of reading skills can already be found in preschool aged children.

In our sample, reading related motivation and precursors of reading skills were moderately correlated. Precursor skills in preschool impacted children's reading related motivation which in turn predicted their reading skills in first grade. While this indirect path had a medium effect size (*d* = 0.60), due to our rather small sample, it turned out to be only marginally significant. Even so other studies typically investigated precursors and reading skills in older children and sometimes measured both variables at one point in time, the covariation we found between precursors and reading skills 1 year later was comparable in strength to previous findings (e.g., Niklas and Schneider, 2012; Oakhill and Cain, 2012; see Swanson et al., 2003, for a review).

We had included parents' highest occupational status (i.e., HISEI) into our analyses, to measure the socioeconomic background of children's families. Consistent with previous research (e.g., Mullis et al., 2012), socioeconomic background and precursors of reading skills in preschool were positively correlated, and even though only marginally significantly so, with a strong effect size (*d* = 0.60).

These findings go beyond previous research in several respects. Reading related motivation has typically been measured and related to reading skills in older groups of children. One possible cause is that particularly large scale assessments require to capture reading motivation via questionnaires which presuppose that the test person can read. In our study, we measured reading related motivation in single session interviews. To assess reading related skills before school entry, we captured precursors of reading skills. In this way, we were able to show that even before the onset of schooling, motivation to read is more pronounced in girls than in boys, is related to precursors of reading skills, and predicts children's future skill development in reading during their first year in school. These findings complement the ones published by Harwardt-Heinecke et al. (2014) who found precursors of reading competence measured in preschool to predict literacy related motivation at the end of first grade. Our findings are also consistent with the ones from a study by Lepola (2004) who found that particularly boys with poor precursors of reading competence in preschool showed a negative motivational trajectory toward literacy related activities and poor reading skills when followed up until the end of first grade. The only study we are aware of which measured reading related motivation via children's self-report and captured precursors of reading skills in children comparable in age to our sample was conducted by Sperling et al. (2013). These authors' reading motivation measure turned out, however, to be unrelated to children's reading related skills. Accordingly, to our knowledge our study is first to show that reading related motivation as described by preschool aged children coincides with strong reading related precursor skills which and predicts reading skills at the end of their first year in school.

Our results also broaden previous work in that we found gender differences in precursors of reading skills in preschool aged children. While some of the studies investigating such precursors in children of this age group found gender differences favoring girls (Camarata and Woodcock, 2006; Below et al., 2010; Lundberg et al., 2012; Wolter et al., 2014), other studies found the genders not to differ in their reading related precursor skills (Fröhlich et al., 2010; Niklas and Schneider, 2012). Future studies need to further investigate under which circumstances gender differences do or do not emerge in precursor skills measured during the preschool years.

### Limitations of our study

HISEI-scores for the families having participated in our study were somewhat higher than previous studies had found in representative samples (e.g., Klieme et al., 2010). While the preschools participating in our studies had randomly been chosen from a list including all 1100 Berlin preschools and while we randomly selected a different one whenever a preschool refused to participate, we cannot rule out that preschools from more affluent districts of the city were more likely to participate. This may have restricted the range of HISEI-scores of the families whose children were included into our study. While our sample may thus not be representative for the Berlin population, we have no reason to suspect that our findings—the interrelatedness between reading related motivation and reading skills, or the impact of preschool teachers' gender role attitudes on children's reading related motivation—would not have turned out, had our sample included more children from families with comparably lower socioeconomic backgrounds. On the contrary, given the slightly limited variance in our HISEI-data, it can be assumed that our findings would have been even stronger, had our sample included more children from families with low HISEI-scores.

In our study we measured preschool teachers' gender role attitudes via self-report, suggesting that a teacher with traditional beliefs more likely deploys gender typing socialization practices in her group of preschool children than a teacher with an egalitarian view on gender roles. Future studies certainly need to substantiate this claim by including variables more directly reflecting teachers' everyday practices, preferably observational data. Also, future studies should include measures of children's reading related gender stereotypes. Such data would allow for a more direct test of the assumption that—via the above described socialization practices—teachers' traditional gender role attitudes impact boys' and girls' reading related motivation and skill development by nourishing children's gender stereotype that “reading is for girls.”

### Conclusions

The findings of our study suggest that preschool teachers' gender role attitudes can have long-term consequences for boys' reading related skill development: Boys who had been with a preschool teacher endorsing traditional gender attitudes were less motivated to learn to read, with motivation in turn predicting their future skill development. In this way, preschool teachers' gender role attitudes operate in a self-fulfilling manner.

In order to improve boys' reading attainments in school, preschool teachers (and school teachers, whom we did not investigate in our study though) should be sensitized that they can contribute to a gender fair learning environment by carefully monitoring their own views on the gender roles, to make sure they do not reinforce gender typed attitudes and behaviors. There is first evidence that gender role attitudes may even be changed systematically by education classes or training (Erden, 2009; Lucier-Greer et al., 2012).

## Funding

This research was supported by a grant from the Deutsche Forschungsgemeinschaft allocated to the last author, BH (HA 2381/8-1, HA 2381/8-2).

### Conflict of interest statement

The authors declare that the research was conducted in the absence of any commercial or financial relationships that could be construed as a potential conflict of interest.

## Footnotes

^1^This study was carried out in accordance with the recommendations of the ethical guidelines of the DGPs (German Psychological Society), as approved of by the ethics committee of the DGPs on December 13, 2004.

## Appendix

Items of the scale “reading related motivation” measured in preschool (t1):

1. How much do you like to learn new rhymes or poems or songs?
2. There are both simple, short poems and songs and some that are longer and more difficult. How much do you like to learn difficult poems and songs too?
3. How much do you like to cite poems or to sing songs without someone helping you?
4. How much to you like someone reading stories to you?
5. How much do you like to tell stories? For example about things that you have experienced, or about something that you have seen on TV?
6. How much do you like to write or draw alphabetic characters?
7. How much do you like to write alphabetic characters without someone helping you?
